# Classification and functional analysis of disulfidptosis‐associated genes in sepsis

**DOI:** 10.1111/jcmm.70020

**Published:** 2024-10-14

**Authors:** Simeng He, Xiangxin Zhang, Zichen Wang, Qingju Zhang, Yu Yao, Jiaojiao Pang, Yuguo Chen

**Affiliations:** ^1^ Department of Emergency Medicine Qilu Hospital of Shandong University Jinan China; ^2^ Shandong Provincial Clinical Research Center for Emergency and Critical Care Medicine, Institute of Emergency and Critical Care Medicine of Shandong University, Chest Pain Center Qilu Hospital of Shandong University Jinan China; ^3^ Key Laboratory of Emergency and Critical Care Medicine of Shandong Province, Key Laboratory of Cardiopulmonary‐Cerebral Resuscitation Research of Shandong Province, Shandong Provincial Engineering Laboratory for Emergency and Critical Care Medicine, Shandong Key Laboratory: Magnetic Field‐free Medicine and Functional Imaging Qilu Hospital of Shandong University Jinan China; ^4^ NMPA Key Laboratory for Clinical Research and Evaluation of Innovative Drug Qilu Hospital of Shandong University Jinan China; ^5^ The Key Laboratory of Cardiovascular Remodeling and Function Research, Chinese Ministry of Education, Chinese Ministry of Health and Chinese Academy of Medical Sciences, The State and Shandong Province Joint Key Laboratory of Translational Cardiovascular Medicine Qilu Hospital of Shandong University Jinan China

**Keywords:** bioinformatics, disulfidptosis, gene expression, immune infiltration, sepsis

## Abstract

Sepsis represents a critical condition characterized by multiple‐organ dysfunction resulting from inflammatory response to infection. Disulfidptosis is a newly identified type of programmed cell death that is intimately associated with the actin cytoskeleton collapse caused by glucose starvation and disulfide stress, but its role in sepsis is largely unknown. The study was to adopt a diagnostic and prognostic signature for sepsis with disulfidptosis based on the differentially expressed genes (DEGs) between sepsis and healthy people from GEO database. The disulfidptosis hub genes associated with sepsis were identified, and then developed consensus clustering and immune infiltration characteristics. Next, we evaluated disulfidptosis‐related risk genes by using LASSO and Random Forest algorithms, and constructed the diagnostic sepsis model by nomogram. Finally, immune infiltration, GSVA analysis and mRNA‐miRNA networks based on disulfidptosis‐related DEGs were screened. There are five upregulated disulfidptosis‐related genes and seven downregulated genes were filtered out. The six intersection disulfidptosis‐related genes including LRPPRC, SLC7A11, GLUT, MYH9, NUBPL and GYS1 exhibited higher predictive ability for sepsis with an accuracy of 99.7%. In addition, the expression patterns of the critical genes were validated. The study provided a comprehensive view of disulfidptosis‐based signatures to predict the prognosis, biological features and potential treatment directions for sepsis.

## 1 INTRODUCTION

Sepsis is recognized as a major global health burden with 48.9 million new cases and 11 million deaths each year.^1^, ^2^ Despite the mortality from sepsis has fallen in recent decades due to advances in treatments including early recognition and resuscitation, infection control and proper antibiotic therapy, and organ support, but representing approximately 20% of all death worldwide.^3^, ^4^ Thus, a more thorough understanding of the pathogenesis and a new therapeutic strategy with high efficiency are urgently needed.

Oxidative stress‐induced programmed cell deaths such as apoptosis, pyroptosis, necroptosis, ferroptosis and autophagic cell death have been notoriously proposed as mechanisms involved in multi‐organ failure during sepsis.^5^, ^6^, ^7^, ^8^ Various types of programmed cell death display unique morphological and biological characteristics but are interrelated.^9^, ^10^, ^11^ Nevertheless, the host response has not been completely elucidated.

Disulfidptosis is a novel form of programmed cell death in which intracellular cystine accumulation promotes disulfide stress and leads to cell death.^12^, ^13^ More specifically, when SLC7A11^hi^ cells are in glucose starvation, massive disulfide bonds with the actin cytoskeleton, presenting collapses of the cytoskeleton networks and cell death.^12^, ^14^ Researchers have found that targeting disulfidptosis might be a potential therapeutic strategy for cancer treatment.^14^ Persistent host defence of sepsis results in extensive metabolic disarray, whether and how disulfidptosis plays a role during sepsis remains unknown.

In the present study, expression profiling from patients with sepsis and control sample were used to identify the significantly differential genes, then a comprehensive and detailed evaluation of disulfidptosis‐related genes in sepsis were analysed including cluster analysis, immune infiltration, risk signature assessment and nomogram to screening the characteristics of disulfidptosis during sepsis and predict the risk of sepsis. Furthermore, the identified genes were verified in a murine sepsis model. Therefore, the identification of disulfidptosis differential expressed genes and immune infiltration in sepsis will improve our knowledge about the molecular mechanism of sepsis and offer new insights for sepsis diagnosis and treatment.

## 2 MATERIALS AND METHODS

### 2.1 Data acquisition

Three sepsis‐related RNA‐seq transcriptomic datasets and Gene Expression Omnibus (GEO) (http://www.ncbi.nlm.nih.gov/geo) database were used to obtained corresponding clinical data. The GSE28750, GSE57065 and GSE95233 were obtained from GPL570 platform, and comprised a total of 89 sepsis samples and 67 health people samples. The normalized data were further processed by the ‘sva’ R package (R.4.2.2). Disulfidptosis‐related genes were accessible through literature mining, including TLN1, ACTB, MYH9, GLUT, NCKAP1, FLNB, SLC3A2, GYS1, FLNA, NDUFA11, RAC1, RPN1, NDUFS1, SLC7A11, NUBPL, LRPPRC and OXSM.^12^

### 2.2 Disulfidptosis‐related differential expression gene analysis

The ‘Limma’ R package was utilized to identify the differentially expressed genes (DEGs) related to disulfidptosis in mRNA between individuals with sepsis and healthy people. Genes with a |logFC|>1.0 and *p*‐values < 0.05 were considered statistically significant.

### 2.3 NMF consensus clustering analysis

In this study, we used non‐negative matrix factorization (NMF) to classify disease samples. NMF is an unsupervised machine learning algorithm suitable for extracting meaningful features from high‐dimensional data.^15^ The basic principle is to decompose the original data matrix into the product of two non‐negative matrices, thereby revealing the underlying structure of the data. After batch correction, GSE28750, GSE57065 and GSE95233 were clustered differently through the ‘NMF’ package by obtaining Disulfidptosis‐related DEGs. The number of clusters (K) from 2 to 10 were tested by running 10 iterations per K, and the optimal K number was eventually determined in accordance with silhouette, consensus, as well as cophenetic.

### 2.4 Analysis of immune infiltration in sepsis

To analyse the heterogeneity of immune cell infiltration, the algorithm of cell type identification by Estimating Relative Subsets of RNA Transcripts (CIBERSORT) was employed which revealed the detail of the abundances of 22 immune cell subtypes. Single sample gene set enrichment analysis (ssGSEA) was further performed to determine the infiltration levels of various immune cells and the ability of immune response.

### 2.5 Consensus clustering

Cluster analysis of sepsis datasets was performed using the R package ‘ConsensusClusterPlus’ to distinguish immune subtypes and identify the key genes related to disulfidptosis. The optimal number of clusters (K) was determined based on cumulative distribution function (CDF) curves, consensus scores and consensus matrix. Principal component analysis (PCA) was subsequently used to assess patterns associated with disulfidptosis.

### 2.6 Development and validation of a nomogram diagnosis model in sepsis

Sepsis‐associated diagnostic variables were screened by LASSO regression and random‐forest algorithm, and the intersection were identified key genes to construct a disease feature map. The predictive value of the key genes in sepsis was evaluated by the receiver operating characteristic curve (ROC) and expressed as the area under the ROC curve (AUC) calculated by the ‘pROC’ R package. Moreover, the ‘rms’ package in R was used to plot a nomogram for predicting sepsis. The prediction performance was evaluated using calibration curves, decision curve analysis (DCA), area under curve (AUC) and clinical impact curve.

### 2.7 Gene set variation analysis

Gene set variation analysis (GSVA) is one of non‐parametric and unsupervised algorithm, which was used to assess the enrichment of ChIP‐seq data and transcriptomic gene sets. Pathway enrichment scores were performed by the ‘GSVA’ package in R.

### 2.8 Construction of regulatory networks

miRNA with potential regulatory relationships with intersection genes between sepsis and disulfidptosis were screened by the Encyclopedia of RNA Interactomes portal (ENCORI, https://rna.sysu.edu.cn/encori/index.php) and the results were visualized with Cytoscape (3.9.1).

### 2.9 Experimental design

C57BL/6J male mice (aged 8 weeks) were purchased from Vital River Laboratory Animal Services Center (Beijing, China) and maintained in standard animal holding room at the experimental animal center of Shandong university Qilu Hospital. Mice were randomly divided into sham (Ctrl) and sepsis (LPS) groups (*n* = 10), and the mice were administered with sterile physiological saline and LPS (15 mg/kg, Sigma, #L2630) via intraperitoneal injection for 12 h. Animals experiments procedure are compliance with the guide for the ethics committee of Shandong university Qilu Hospital (approval number: DWLL‐2022‐136).

### 2.10 Quantitative real‐time reverse transcriptase polymerase chain reaction

Total RNA from tissues were isolated using TRIzol method (Invitrogen, Carlsbad, CA) following the manufacturer's manuals. 1 μg RNA was converted into cDNA using Hifair® III 1st Strand cDNA Synthesis Mix (YEASEN, Shanghai, China) under the standardized procedure: 25°C for 5 min, 55°C for 15 min and 85°C for 5 min. qPCR analysis was performed using Hieff UNICON® Universal Blue qPCR SYBR Green Master Mix (YEASEN, Shanghai, China). Applied Biosystems 7500 was performed for pre‐degeneration at 95°C for 2 min, followed by 40 thermal cycles consisting of denaturing for 10 s at 95°C and annealing for 32 s at 60°C. The 2^−ΔΔCt^ method was calculated to determine the relative mRNA expression of target genes including LRPPRC, SLC7A11, MYH9, NUBPL, GYS1, GLUT1, and the primers were listed in Table S1.

### 2.11 Western blotting analysis

Proteins were extracted from heart, liver, lung, kidney tissues. The bicinchoninic acid (BCA) reagent (Beyotime, Shanghai, China) was used to determine protein concentrations. Twenty microgram samples were loaded on 10% SDS‐PAGE for electrophoresis and then transfer to a PVDF membrane, and then incubated with primary antibodies for SLC7A11 (1:1000, Cell Signalling Technology, #12691) and GAPDH (1:5000, Proteintech, #60004‐1‐Ig) at 4°C overnight. After washed with TBST five times, the blots were infiltrated with appropriated secondary antibody (1:5000, Jackson, 111‐035‐003) for 1–2 h at room temperature. Finally, the blots were visualized by enhanced chemiluminescence system (Merck‐Millipore, USA, #WBKLS0500) and analysed using ImageJ system.

### 2.12 Histological analysis

The collected tissues were fixed with 4% paraformaldehyde for over 48 h followed by routine dehydration, transparency, wax immersion and embedding with 4 μm sections. After haematoxylin/eosin (H&E) staining, dehydrated and transparently sealed, slices were scanned by digital slice scanner (Olympus VS200).

### 2.13 Immunofluorescence

Slices were fixed with 4% paraformaldehyde for 10 min and permeabilized with 0.1% Triton X‐100 for 10 min. After blocking with 5% goat serum for 30 min at 37°C, slices were incubated overnight with SLC7A11 antibody (1:250, Cell Signalling Technology, #12691). Then, slices were using PBS solution and the secondary antibody was incubated for 30–60 min at 37°C in the dark, followed by DAPI incubation to labelled the nuclei.

### 2.14 Statistical analysis

Values are expressed as means ± SEM (standard error of mean). Comparisons between two groups were performed using unpaired *T*‐test by Graph Prism 8.0 software (GraphPad, La Jolla, CA, USA). *p*‐values of less than 0.05 were indicated statistically significant (*p < 0.05; **p < 0.01; ***p < 0.001).

## 3 RESULTS

### 3.1 Screening for DEGs and landscape of the infiltrated immune cells in sepsis

**FIGURE 1:**
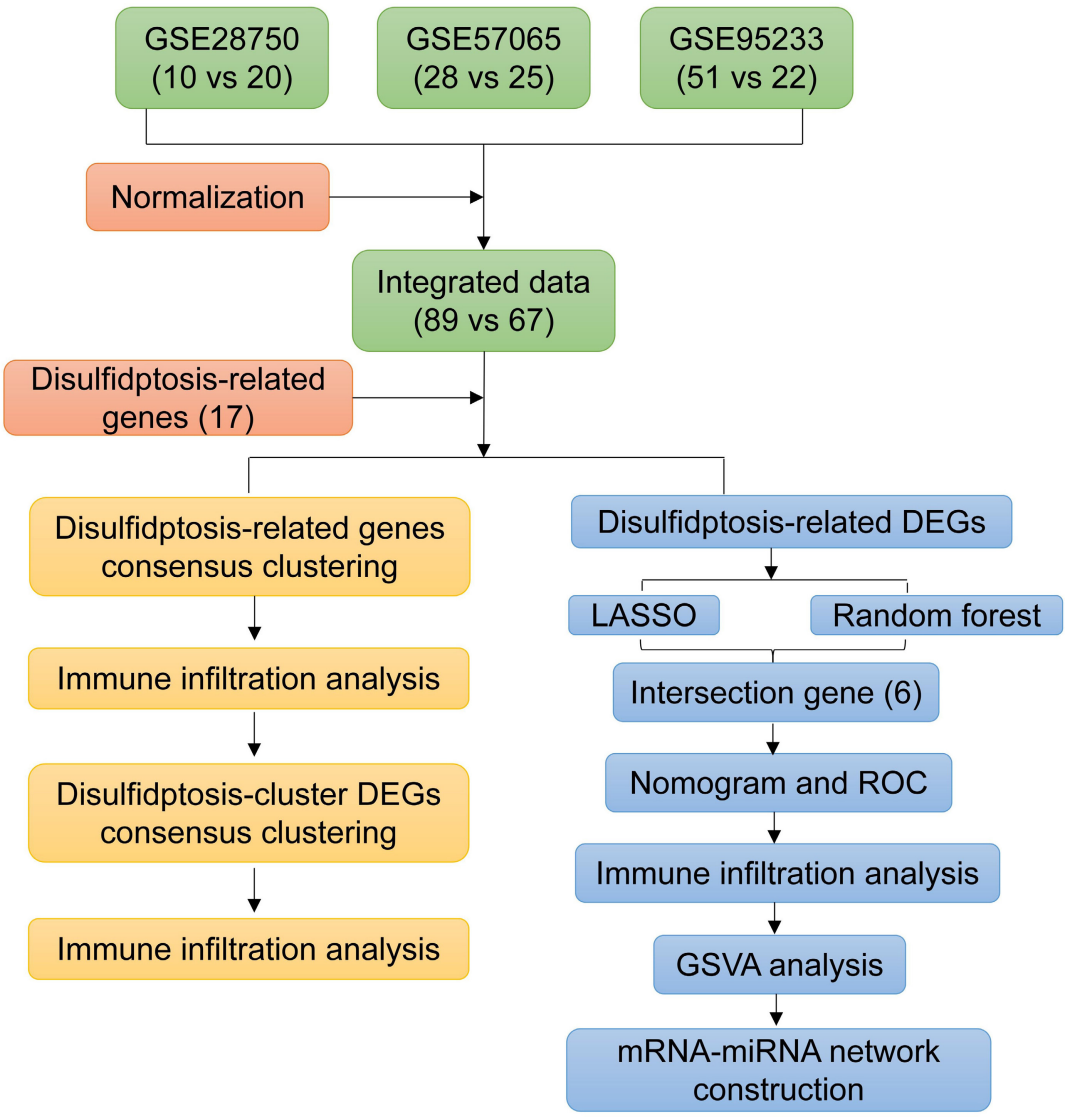
Flow chart.

**FIGURE 2:**
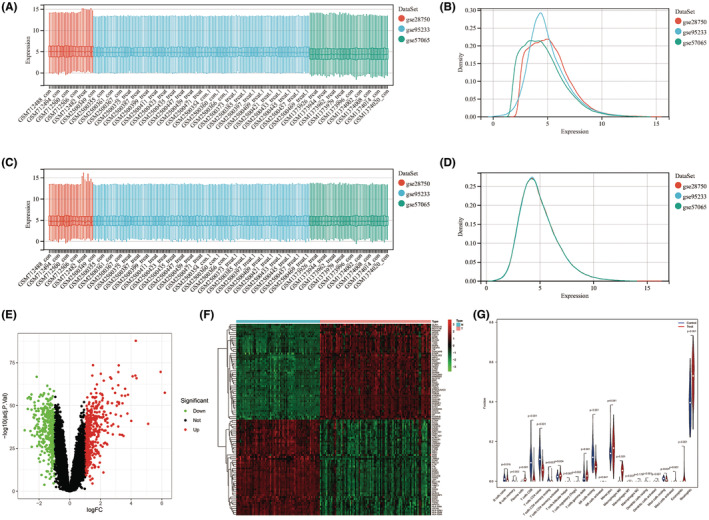
Visualization of three sepsis datasets before and after batch and normalization. (A, B) Datasets before batch and normalization. (C, D) Datasets after batch and normalization. (E) Volcano plot of sepsis‐related DEGs in which red nodes indicate upregulated, green nodes indicate downregulated, and black nodes indicate genes are not DEGs. (F) Heat map of sepsis‐related DEGs expressions: blue (type N) indicates normal samples, pink (type T) indicates disease sample, red and green indicates high and low expression, respectively. (G) Violin chart showing differences in immune infiltration between sepsis patients and healthy people. *p < 0.05, **p < 0.01, ***p < 0.001.

The study workflow is illustrated schematically in Figure 1. The sepsis‐related RNA‐seq and microarray datasets GSE57065, GSE95233 and GSE28750 were obtained from the GEO database. The summarized information of the sepsis‐related datasets was shown in Table S2. The particulars dataset after removing the batch effect were displayed in Figure 2D. The DEGs between sepsis patients and healthy controls were then analysed using the ‘Limma’ R package. Totally, 838 DEGs were identified, of which 447 were upregulated and 383 were downregulated genes (Figure 2E,F).

Dysregulated immune response is the most significant pathologic feature of sepsis.^16^, ^17^ The fraction of different subtypes of infiltration immune cells among sepsis and healthy groups was assessed by the CIBERSORT algorithm.^18^ Significant differences were observed between the 19 immune cells including neutrophils, monocytes, M0 macrophages, CD8^+^ T cells, resting NK cells, naïve CD4^+^ T cells, etc., and the detailed presentation is shown in Figure 2G.

### 3.2 Identification of disulfidptosis‐related DEGs between sepsis and control

**FIGURE 3:**
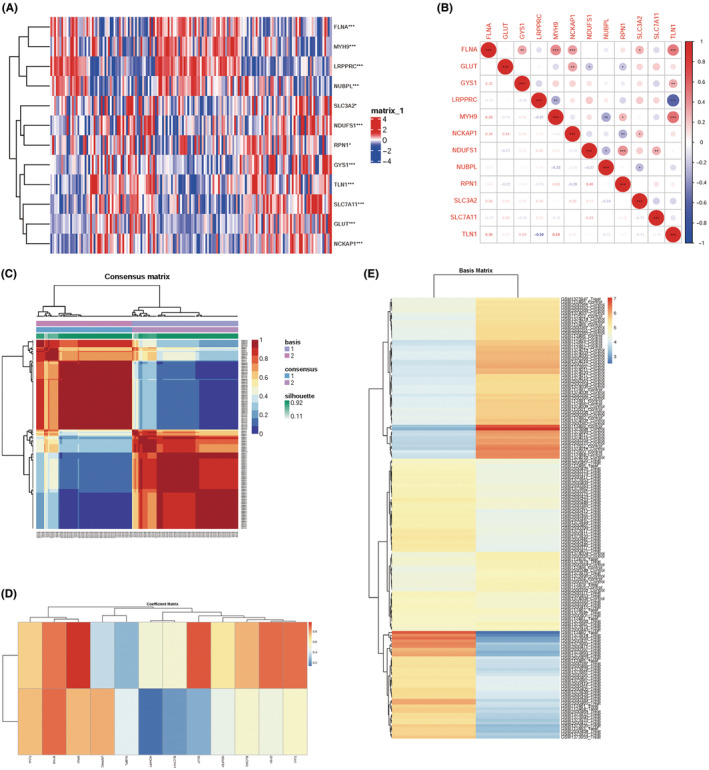
Differential expression, correlation and NMF clustering of genes associated with disulfide poisoning in sepsis. (A) Heatmap display the expression of 12 disulfidptosis‐related DEGs. (B) The relative positions of the 12 disulfidptosis‐ related DEGs. (C) NMF consensus clustering for *k* = 2. (D) Heatmap of Coefficient matrix. (E) Heatmap of Basis matrix. *p < 0.05, **p < 0.01, ***p < 0.001.

To screen the interrelationship between sepsis and disulfidptosis, we first extracted disulfidptosis‐related genes from the latest literature and filtered out five upregulated genes (FLNA, LRPPRC, SLC7A11, NUBPL and RPN1) and seven downregulated genes (GLUT, GYS1, MYH9, NCKAP1, NDUFS1, SLC3A2 and TLN1) showed the differential expression between sepsis and disulfidptosis (Figure 3A). Moreover, the correlation between differential genes showed that most genes were cross‐correlation. (Figure 3B).

### 3.3 Identification of disulfidptosis‐associated cluster and analysis of immune microenvironment

**FIGURE 4:**
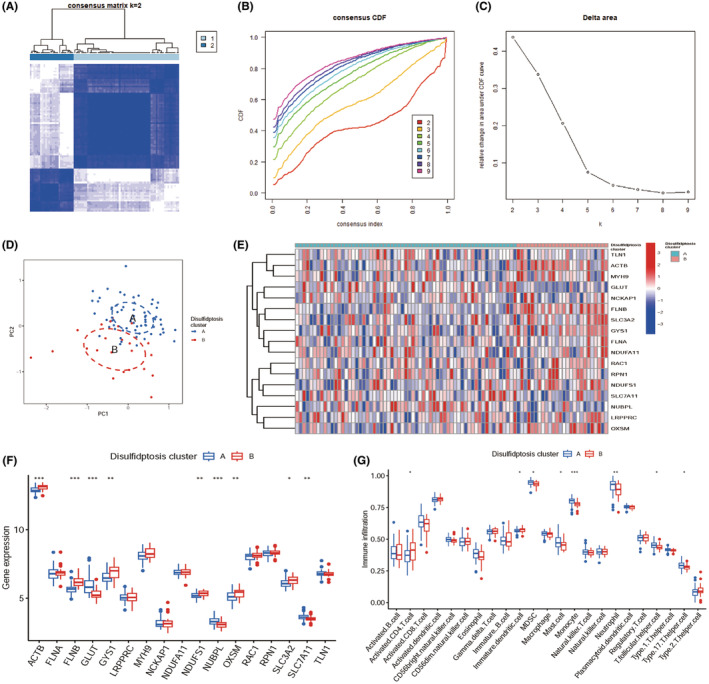
Identification of disulfidptosis subtypes and immune signature. (A) Disulfidptosis‐associated cluster. (B) Representative cumulative distribution function (CDF) curve. (C) Representative CDF delta area curve. (D) A principal component analysis (PCA) visualization of the cluster distributions. (E) Two disulfidptosis clusters of 17 disulfidptosis‐related genes were represented in the heatmap. (F) Boxplots showing differences in the expression of 17 disulfidptosis‐related genes between the two disulfidptosis clusters. (G) Boxplots display the comparison of immune infiltration in two disulfidptosis clusters. *p < 0.05, **p < 0.01, ***p < 0.001.

NMF consensus clustering was conducted on the three datasets on the basis of disulfidptosis‐associated DEGs. Following the prompts of consensus and cophenetic, all samples were eventually split into two clusters (Figure 3C), and the heatmap of basis matrix and coefficient matrix were shown in the Figure 3D,E. Based on the disulfidptosis‐related DEGs, the cluster analysis was carried out using the ‘ConsensusClusterPlus’ R package (Figure 4A–C). The cluster effect was optimal when sepsis patients were divided into two clusters A1 and A2, of which the internal consistency and stability were reliable. Principal component analysis (PCA) revealed that the two different clusters can be distinguished completely by the 17 disulfidptosis‐related genes (Figure 4D).

The heat map for the differentially disulfidptosis‐related genes among the clusters was then constructed to show that GLUT, NUBPL and SLC7A11 were significantly upregulated in cluster A, while ACTB, FLNB, GYS1, NDUFS1, OXSM, SLC3A2 were significantly upregulated in cluster B (Figure 4E,F).

After visualized immune cell infiltration, we found that cluster A with a higher infiltration of MDSC, mast cell, monocyte, neutrophil, T follicular helper cell and type 17 T helper cell, whereas cluster B with a higher infiltration of activated CD4^+^ T cell and immature dendritic cell (Figure 4G).

### 3.4 Characterization of cluster based on disulfidptosis‐associated DEGs and immune microenvironment

**FIGURE 5:**
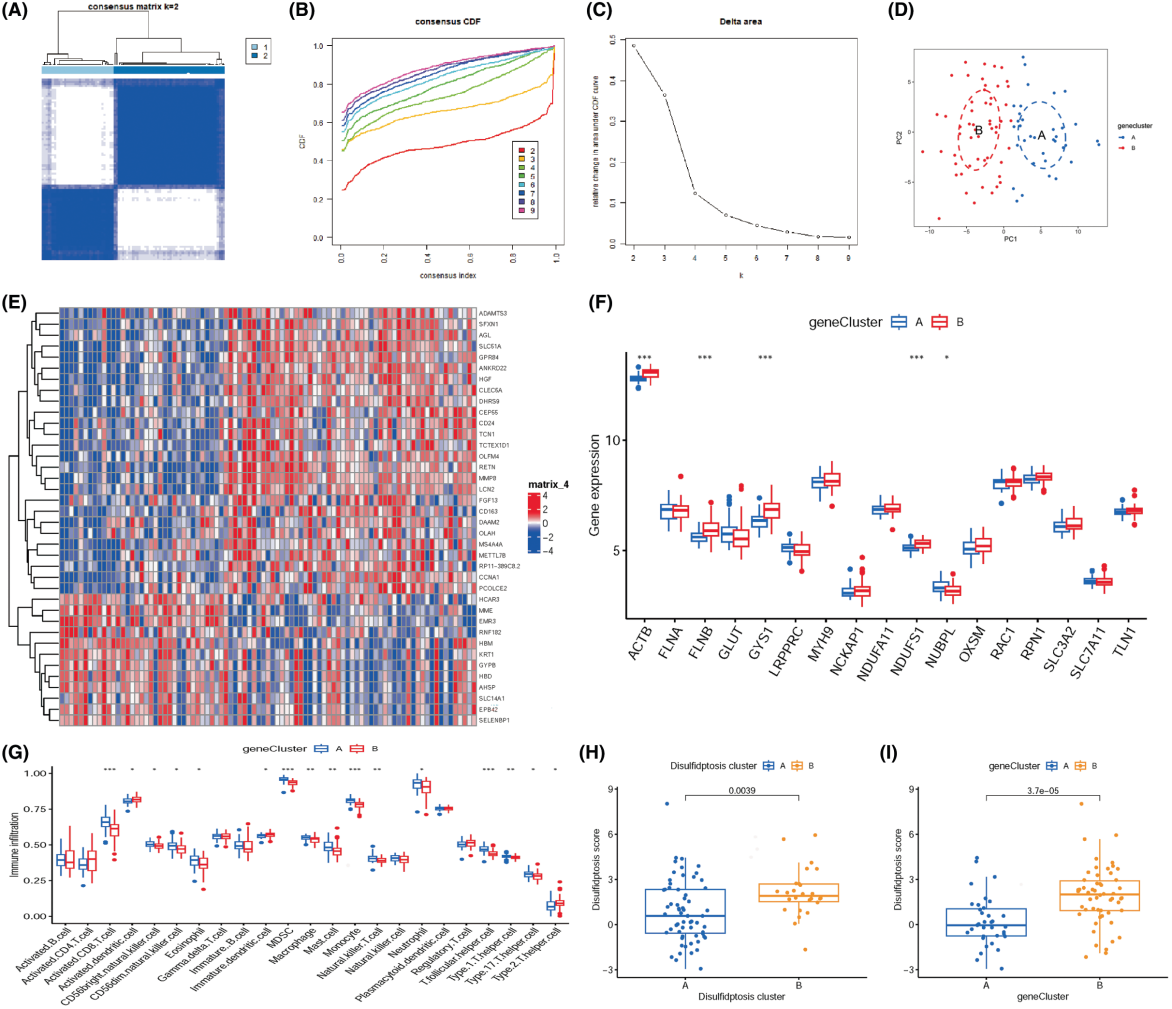
Explore the gene clusters based on the DEGs of disulfidptosis clusters. (A) Based on 38 DEGs, the consensus clustering matrix for *k* = 2. (B) CDF curve. (C) CDF delta area curve. (D) PCA visualization of the distribution of the two gene clusters. (E) Heatmap of the 38 DEGs between disulfidptosis clusters. (F) Boxplots showing the disulfidptosis‐associated DEGs in the two gene clusters. (G) Relative abundance of immune cells between the two gene clusters. (H–I) There are statistically significant differences in the disulfidptosis score both in disulfidptosis clusters (H) and disulfidptosis‐related DEGs cluster (I). *p < 0.05, **p < 0.01, ***p < 0.001.

To further verify the disulfidptosis cluster, DEGs between clusters 1 and 2 were screened and found that 38 DEGs (Table S3). Subsequently, a consensus clustering method was used to group sepsis patients into distinct genomic subtypes based on the disulfidptosis‐associated DEGs (Figure 5A). The consensus cluster CDF curve indicated that the parameter k = 2 is the optimal grouping (Figure 5B,C). PCA analysis suggested that 38 DEGs could distinguish completely two sub‐clusters (Figure 5D,E). Meanwhile, the expressions of ACTB, FLNB, GYS1, NDUFS1 and NUBPL were significantly varied between two clusters A and B (Figure 5F).

We next observed the immune infiltration in the two clusters, the results exhibited that the infiltrating status of 16 immune cells had marked differences, including activated CD8^+^ T cell, activated dendritic cell, CD56 bright natural killer cell, CD56 dim natural killer cell, eosinophil, immature dendritic cell, MDSC, macrophage, mast cell, monocyte, natural killer cell, neutrophil, T follicular helper cell, type 1/2 T helper cell, type 17 T helper cell (Figure 5G). Importantly, MDSC and neutrophils showed higher levels of infiltration.

Furthermore, we calculated the disulfidptosis scores based on the disulfidptosis cluster and disulfidptosis‐related DEGs cluster and found that the scores in A and B have significant differences, which also illustrated that the classification was reliable (Figure 5H,I).

### 3.5 Construction of a disulfidptosis‐related model and a nomogram based on the model

**FIGURE 6:**
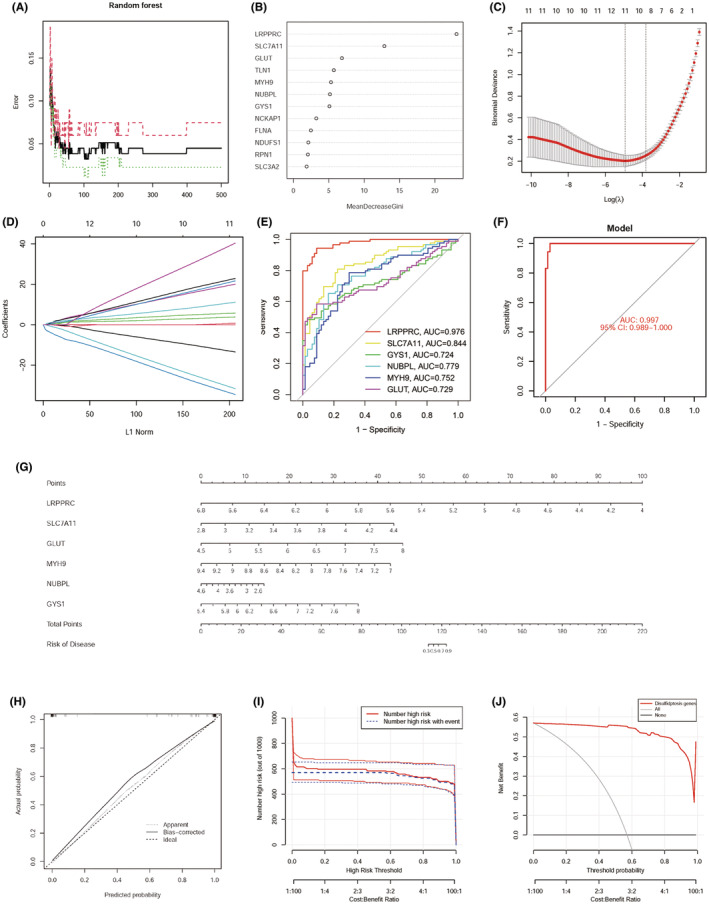
Construction and validation of the least absolute shrinkage and selection operator (LASSO) model and Random Forest (RF) model. (A) The influence of the number of decision trees on the error rate. (B) Results of the Gini coefficient method in the random forest classifier. (C, D) Screening of disulfidptosis‐related differentially expressed genes (DEGs) signature using the LASSO algorithm. (E) The ROC curve of the mRNA in the model (LRPPRC, SLC7ALL, GYS1, NUBPL, MYH9, GLUT). (F) The AUC of the constructed model (AUC = 0.997, 95% CI:0.989–1.000). (G) The ordinary nomogram for the joint diagnosis of sepsis is based on LRPPRC, SLC7A11, GLUT, MYH9, NUBPL, and GYS1. (H) Calibration curve for nomogram validation. (I, J) The clinical impact curve and decision curve analysis are based on the nomogram model.

To determine candidate genes for disulfidptosis predicting the occurrence of sepsis, we constructed the LASSO regression model and RF algorithm.^19^ The RF model provided the initial ranking of variables importance according to the mean decrease Gini index, and we acquired seven genes that scored larger than five including LRPPRC, SLC7A11, GLUT, TLNI, MYH9, NUBPL and GSY1 (Figure 6A,B). LASSO regression analysis showed that 11 disulfidptosis‐related genes (including FLNA, GLUT, GYS1, LRPPRC, MYH9, NCKAP1, NDUFS1, NUBPL, RPN1, SLC3A2 and SLC7A11) were associated with sepsis (Figure 6C,D). In total, six intersection genes LRPPRC, SLC7A11, GLUT, MYH9, NUBPL and GYS1 were seen by both models. Subsequently, the predictive model was constructed using the 6 intersection genes and evaluation of diagnostic accuracy was applied using ROC analysis. The results showed that all six genes had higher predictive values and the predictive model achieves high accuracy of 99.7% (Figure 6E,F).

By using the above six key signatures, the nomogram was constructed to better predict sepsis risk which the higher scores corresponded to a high risk of sepsis (Figure 6G). Then, the calibration curve also demonstrated the accurate predictive ability of this nomogram and the results indicated that the bias‐correct curve matches well with the ideal curve (Figure 6H). Moreover, both the clinical impact curve and decision curve analysis (DCA) suggested that the risk model had significant predictive power (Figure 6I,J). Specifically, the red line was always above the grey line which indicated that the decision based on the nomograms can benefit sepsis patients.

### 3.6 Landscape of the infiltrated immune cells in disulfidptosis

**FIGURE 7:**
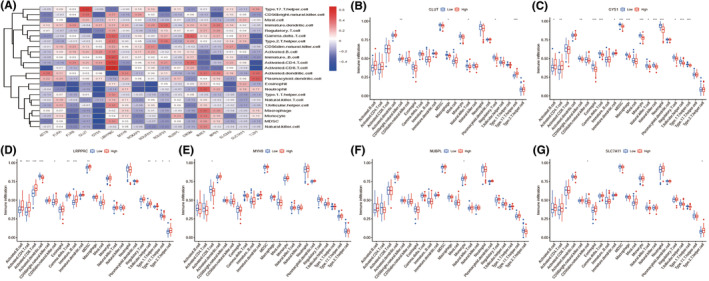
Immune infiltration analysis of the disulfidptosis‐related genes. (A) Heatmap showing the correlation between disulfidptosis‐related genes and the different immune cells. (B–G) The expression level of genes in the signature and the infiltration abundance of immune cells. *p < 0.05, **p < 0.01, ***p < 0.001.

Relationship between disulfidptosis‐related characteristics and the immune cells was observed and exhibited in a complete heatmap in Figure 7A. The expressions of these six DEGs (LRPPRC, SLC7A11, GLUT, TLNI, MYH9, NUBPL and GSY1) in different infiltrated immune cells were evaluated in further depth (Figure 7B–G).

### 3.7 Functional insights into the disulfidptosis‐related genes

**FIGURE 8:**
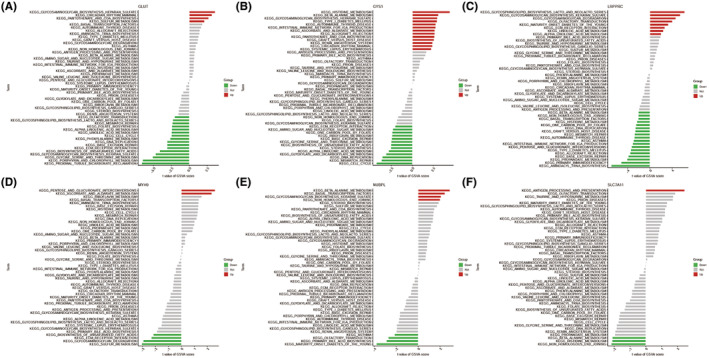
GSVA analysis showing the different KEGG pathways related to six model genes. (A) GLUT. (B) GYS1. (C) LRPPRC. (D) MYH9. (E) NUBPL. (F) SLC7A11. Green indicates down‐regulated; red indicates up‐regulated; gray indicates not.

To further explore the biological functions and potential signalling pathways involved in these disulfidptosis‐related characteristics, we performed GSVA analysis (Gene Set Variation Analysis) for the overlapping GEGs as shown in Figure 8. For example, GLUT is upregulated in various biological processes such as ‘glycosaminoglycan biosynthesis heparan sulfate’, ‘circadian rhythm mammal’, ‘pantothenate and coa biosynthesis’ and ‘sulfur metabolism’, whereas downregulated in 16 biological processes such as ‘olfactory transduction’, ‘glycosphingolipid biosynthesis lacto and neolacto series’, ‘mismatch repair’, ‘folate biosynthesis’, ‘alpha linolenic acid metabolism’, ‘linoleic acid metabolism’, ‘cell cycle’, ‘phenylalanine metabolism’, ‘DNA replication’, ‘base excision repair’, ‘ECM receptor interaction’, ‘biosynthesis of unsaturated fatty acids’, ‘glycine serine and threonine metabolism’, ‘porphyrin and chlorophyll metabolism’ and ‘proximal tubule bicarbonate reclamation’.

### 3.8 Construction of regulatory networks at target miRNAs

**FIGURE 9:**
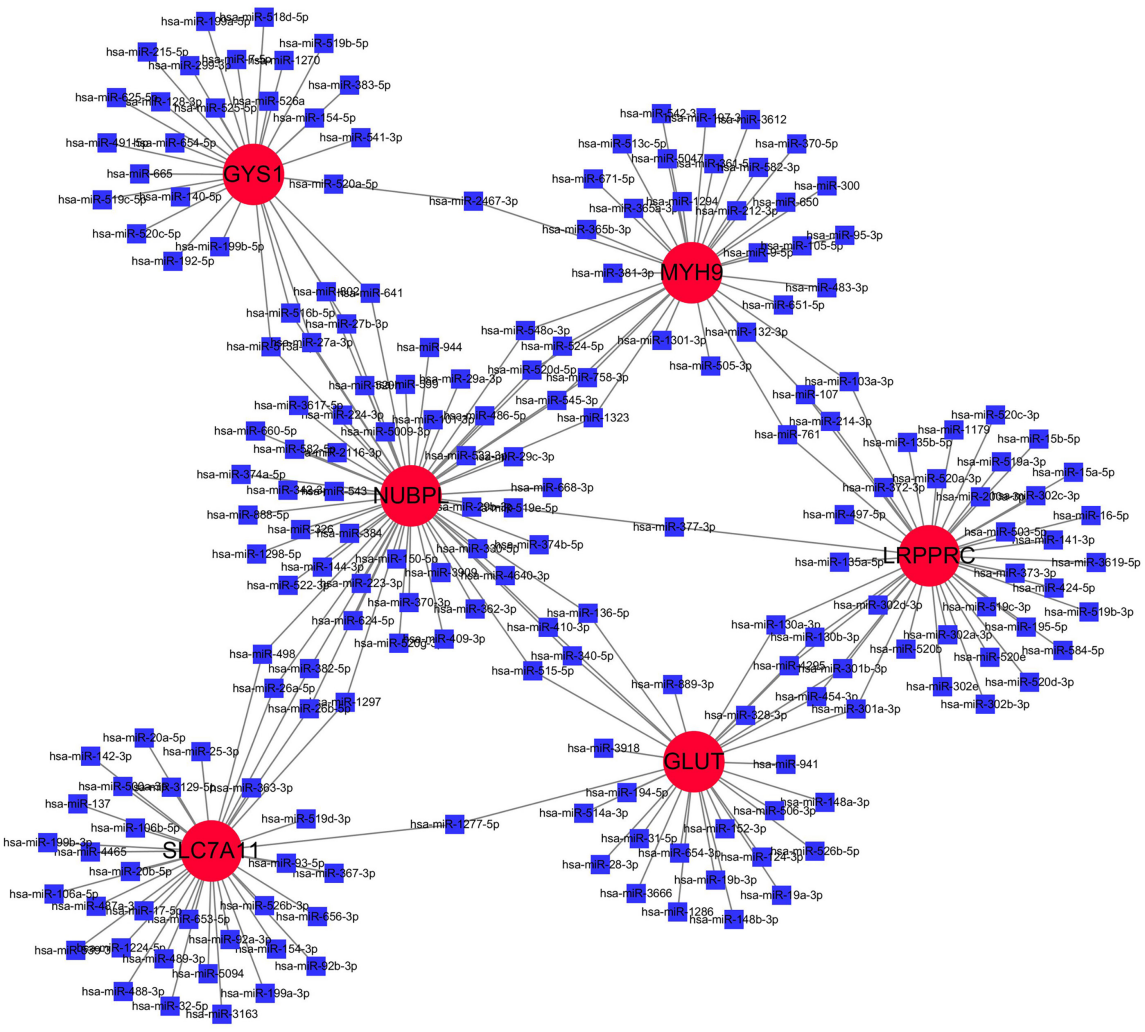
MRNA‐miRNA regulatory network with potential regulatory relationships with six genes in the model.

To obtain substantive changes happening at the transcript profiles and gain deeper insights into the overlapping DEGs, a network‐based method was adopted to decode the regulatory miRNAs. Thirty miRNAs were figured out from the ENCORI website (http://starbase.sysu.edu.cn/index.php) that could potentially be regulated with hub genes. The blue squares represented miRNAs and the red circles represented DEGs (Figure 9). The meaningful miRNAs could provide novel directions for targeted therapy.

### 3.9 Validation of disulfidptosis in sepsis

**FIGURE 10:**
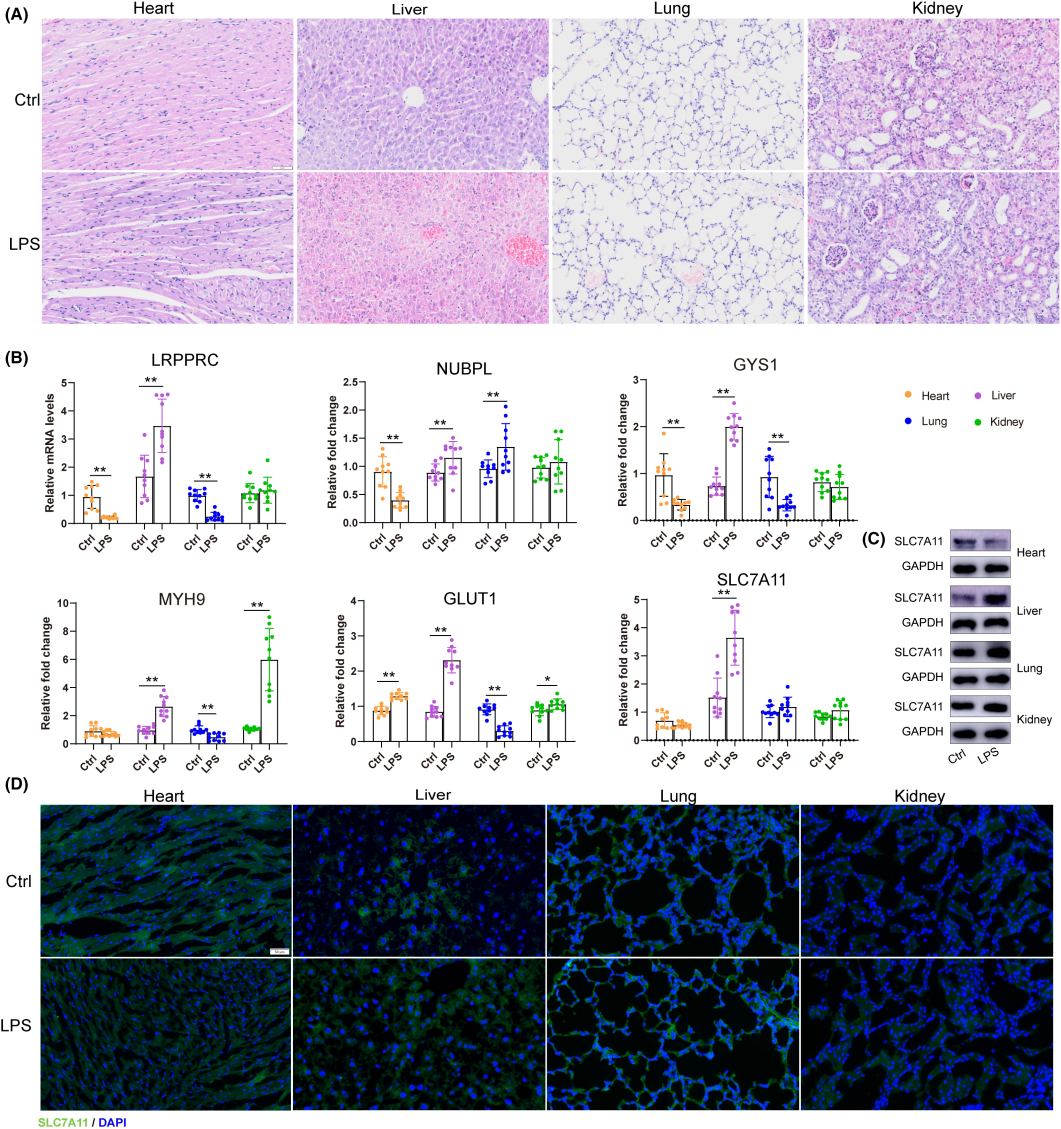
Validation of murine sepsis model. (A) Represented HE staining among control and LPS groups. (B) The mRNA expression levels of LRPPRC, NUBPL, GYS1, MYH9, GLUT1, and SLC7A11 in heart, lung, liver, and kidney. (C) Western blot for SLC7A11. (D) Representative immunofluorescence images for SLC7A11. *p < 0.05, **p < 0.01.

To verify the screening results, LPS administration intraperitoneally was to establish a murine model of sepsis. Representative images of HE staining revealed the organs including heart, liver, lung and kidney pathological changes (Figure 10A). The results of PCR suggested that the mRNA expression levels LRPPRC, NUBPL, GYS1, MYH9, GLUT1 and SLC7A11, with different trends in the alterations among different tissues (Figure 10B). Moreover, as a switch protein of disulfidptosis, SLC7A11 was detected by western blot and immunofluorescence analysis. SLC7A11 protein expressions were significantly higher in LPS group in liver, lung, kidney but downregulated in the heart (Figure 10C,D). Collectively, these results preliminary presented the alterations of disulfidptosis‐related DEGs in sepsis.

## 4 DISCUSSION

Sepsis represents a complex syndrome with dysregulated immune response and high mortality.^1^ With the COVID‐19 pandemic worldwide, some critically ill or severe patients rapidly progressed to multiple organ dysfunction syndrome (MODS) that fit the diagnostic criteria of sepsis, of which the prevention and treatment of sepsis remains a clinical conundrum.^20^, ^21^ Various types of programmed cell death such as apoptosis, necroptosis, autophagy, ferroptosis and cuproptosis have been proposed as important mechanisms participating in sepsis.^7^, ^22^ Targeting such a death cascade throughgenetic or pharmacological interventions could regulate the disease severity and progression, and a substantial body of research has advanced greatly over the past decades. Recently, Liu et al investigated a novel mechanism of death, disulfidptosis, exhibiting disulfide bond increase induced by glucose starvation and cystine accumulation.^12^ As sepsis is correlated with glucose metabolic disorders, we explored whether disulfidptosis are present in sepsis and what potential role it performs.

Disulfidptosis is the process of cell death triggered by disulfide stress and actin cytoskeleton collapse.^6^, ^14^ The disulfide bond formation results from cystine accumulation associated with SLC7A11 cystine–glutamate antiporter insufficient or dysfunction and glucose starvation mediated NADPH depletion.^14^ Interestingly, there was a tight correlation between disulfidptosis and ferroptosis. Ferroptosis is an iron‐dependent programmed cell death driven by lipid peroxidation and cystine‐uptake blocked.^23^, ^24^, ^25^ Cystine/cysteine uptake depends on SLC7A11 from the extracellular environment into cytoplasm.^26^, ^27^ On the one hand, upregulation or activation of SLC7A11 could import supply cystine for glutathione production which needs sufficient NADPH via the pentose phosphate pathway (PPP), and eventually avoid ferroptosis.^14^ On the other hand, SLC7A11^high^ cells starved of glucose could induce disulfidptosis, but the process is not affected by iron or inhibition of fatty acid oxidation.^12^, ^14^ Furthermore, it is well established that widespread oxidative stress is a prominent feature of sepsis that triggers programmed cell death.^28^, ^29^, ^30^ However, these disulfidptosis changes are not prevented by suppressing ROS (reactive oxygen species) production but entirely rely on cystine uptake mediated by SLC7A11.^14^ This is perhaps the biggest difference between disulfidptosis and other types of programmed cell death.

The association between disulfidptosis and sepsis pathophysiology is inextricably linked and also deserves insightful discussion. Numerous studies indicated that glycogen metabolism is important for macrophage‐mediated inflammatory reactions, apart from glycolysis, pentose phosphate pathway and mitochondrial succinate oxidation have been indicated to drive their phenotype.^31^ The changes in glucose metabolism are different at different stages and exerted different effects on sepsis pathophysiological characteristics.^32^, ^33^, ^34^ Additionally, persistent and powerful immune responses perform complex cellular metabolic regulation.^35^, ^36^ Thus, we inferred disulfidptosis might play essential roles during the pathogenesis of sepsis, and sought to understand the signature and predictive power of disulfidptosis in sepsis. In the present study, the expression pattens of disulfidptosis‐related DEGs were verified in various organs. The majority of DEGs were differentially expressed between control and LPS groups, but not the same among organs. This might be because that the different overdose or duration of LPS stimulation led to different severities of impairment of the organs, animal models based on the severity of organ damage could be used to identify the association of DEGs and organ dysfunctions in sepsis.

However, there are also some potential shortcomings in the study. First, we only revealed the correlation between the newly termed form of cell death, and disulfidptosis with sepsis, but did not analyse the association with other programmed cell death. Second, the prognostic disulfidptosis‐signature constructed in our study was based on the public database and lacked clinical data to validate these findings. Future work will explore these details in greater depth.

## 5 CONCLUSION

In summary, our study represented the first comprehensive analyse the role of disulfidptosis‐related genes in sepsis. We screened the consensus clustering and machine‐learn analysis based disulfidptosis, and discussed the immune infiltration and the prognostic potential. Thus, the study will aid in elucidating the pathogenesis of sepsis, and inspire more effective immune and individualized treatment.

## AUTHOR CONTRIBUTIONS

**Simeng He:** Formal analysis (lead); writing – original draft (lead). **Xiangxin Zhang:** Formal analysis (equal). **Zichen Wang:** Writing – review and editing (equal). **Qingju Zhang:** Writing – review and editing (supporting). **Yu Yao:** Formal analysis (supporting). **Jiaojiao Pang:** Conceptualization (equal). **Yuguo Chen:** Conceptualization (equal).

## FUNDING INFORMATION

This study was supported by the Key R&D Program of Shandong Province (2021SFGC0503), State Key Program of the National Natural Science Foundation of China (82030059), National Natural Science Foundation of China (82172127, 81772036, 82072144, 81671952, 81873950, 81873953), National Key R&D Program of China (2020YFC1512700, 2020YFC1512705, 2020YFC1512703, 2020YFC0846600), National S&T Fundamental Resources Investigation Project (2018FY100600, 2018FY100602), Taishan Pandeng Scholar Program of Shandong Province (tspd20181220), Taishan Young Scholar Program of Shandong Province (tsqn20161065, tsqn201812129), Youth Top‐Talent Project of National Ten Thousand Talents Plan, Qilu Young Scholar Program and the Fundamental Research Funds of Shandong University (2018JC011), Natural Science Foundation of Shandong Province (ZR2023QH324).

## CONFLICT OF INTEREST STATEMENT

All authors disclose no conflict of interest.

## Supporting information


Data S1.

## Data Availability

All the datasets presented in the study are available in the GEO database online (accession numbers: GSE28750, GSE57065, and GSE95233).
