# Supplementary material for: Classification and functional analysis of disulfidptosis‐associated genes in sepsis
Source: J Cell Mol Med. 2024 Oct 14;28(19):e70020. doi: 10.1111/jcmm.70020 (PMC11472650; doi:10.1111/jcmm.70020)
Supplement: Supplementary file 1 — Data S1. [file JCMM-28-e70020-s001.docx]

**Supplementary tables**

**Supplementary Table 1** Primer sequences for RT-PCR

| **Gene** | **Forward primer (5’→3’)** | **Reverse primer (5’ →3’)** |
| --- | --- | --- |
| LRPPRC | GACCCGCGCGTTGGC | TCCCTTTTCTCAGCAACGATGG |
| SLC7A11 | ATATCCAGAACACGGGCA | AGGGCTCCAAAAAGTGACAGT |
| MYH9 | ATCTCGTGCTATCCGCCAAG | CGTTGGACAGGAAGCGGTAT |
| NUBPL | GCCAGTTATTAGGCGCTGAG | TCCAACACCACCCTTTCCAG |
| GYS1 | GTCTCTCTGTGTCCTCGCTTC | GTCACCTTCGCCTTCGTCTG |
| GLUT1 | ATCATCGGTGTGTACTGCGG | AGCCAAACACCTGGGCAATA |
| GAPDH | TGTCTCCTGCGACTTCAACA | GGTGGTCCAGGGTTTCTTACT |

**Supplementary** **Table 2** Sepsis datasets information

| **GEO accession** | **Species** | **Number of samples**  (sepsis/control) |
| --- | --- | --- |
| GSE28750 | homo sapien | 10/20 |
| GSE57065 | homo sapien | 28/25 |
| GSE95233 | homo sapien | 51/22 |

**Supplementary Table 3** DEGs between disulfidptosis clusters

| RP11-389C8.2 | SFXN1 | HBD | DAAM2 | MMP8 | SLC51A | GYPB | CEP55 | SLC14A1 | OLFM4 |
| --- | --- | --- | --- | --- | --- | --- | --- | --- | --- |
| RETN | MS4A4A | AGL | MME | EMR3 | GPR84 | KRT1 | SELENBP1 | TCN1 | RNF182 |
| ANKRD22 | HGF | CLEC5A | DHRS9 | EPB42 | HCAR3 | CD24 | LCN2 | PCOLCE2 |  |
| HBM | TCTEX1D1 | ADAMTS3 | CD163 | AHSP | OLAH | METTL7B | CCNA1 | FGF13 |  |
